# A Comparison of Physical Activity and Exercise Recommendations for Public Health: Inconsistent Activity Messages Are Being Conveyed to the General Public

**DOI:** 10.3390/sports12120335

**Published:** 2024-12-04

**Authors:** Vincent J. Dalbo, Michael A. Carron

**Affiliations:** Health, Education, Lifestyle, and Performance (HELP) Laboratory, St. Brendan’s College, Yeppoon, QLD 4703, Australia

**Keywords:** 10,000 steps, guidelines, health promotion, wellness, wellbeing, walking, government

## Abstract

We examined the similarities and differences between government-supported public health activity recommendations from the World Health Organization (WHO), the Centers for Disease Control and Prevention (CDC), the National Health Service (NHS), the Department of Health and Aged Care (DHAC), and one of the most renowned public health activity recommendations, the 10,000 Steps Program. The findings derived from our evaluation suggest a lack of consistency in public health activity recommendations, including the nomenclature used to describe aerobic activity, the amount of time required per week to meet the minimum recommendation for moderate and vigorous activity, and variations in the intensities required to meet aerobic activity recommendations. We also found that moderate-intensity activity (3.0 to less than 6.0 METS) is achieved across the lifespan with normal (i.e., mean), rather than vigorous, walking speeds; this suggests the MET level for moderate-intensity activity may need to be re-examined. The suggested strength activities must also be considered to ensure that the activities maintain or improve strength in the general public. Among the reviewed recommendations, none distinguished between physical activity and exercise, which may contribute to the low levels of exercise participation among the general public. Since exercise is medicine, the most recognized government-supported public health activity recommendations should place a greater emphasis on exercise over physical activity. Moreover, given the low levels of activity in the general public, more care should be given to provide a consistent, clear, and direct message regarding activity recommendations.

**WHAT IS ALREADY KNOWN ON THIS TOPIC**
  - Each year, the number of people living with disease increases, which is largely associated with the general public not meeting recommended activity guidelines.
  - The World Health Organization, Centers for Disease Control and Prevention, National Health Service, Department of Health and Aged Care, and the 10,000 Steps Program provide recommendations for weekly activity to improve the health of the general public.
  - Each year, new initiatives are implemented to increase activity among the general public.
**WHAT THIS STUDY ADDS**
  - Public health activity recommendations do not send a consistent, clear, or direct message to the general public, which may contribute to the low levels of physical activity and exercise performed globally.
  - Activity recommendations to maintain or improve the health of an 18 year old should differ from the recommendations for a 64 year old; therefore, we constructed a theoretical framework for future activity recommendations for adults aged 18 to <45 years, 45 to <65 years, and ≥65 years.
  - Public health activity recommendations targeting the general public must ensure the recommendations maintain or improve the health of the general public, not just the unhealthiest individuals in this cohort.
  - The moniker ‘exercise is medicine’ has become a battle cry for healthcare professionals, but none of the public health activity recommendations examined in this review make the distinction between physical activity and exercise; this may contribute to the low levels of exercise performed in the general public.

## 1. Introduction

Each year, the issue of poor health amongst the general public becomes more problematic, resulting in economic and personal losses. The economic impact of poor health can be quantified by increasing healthcare costs [1,2] and the loss of work productivity [3,4,5]; meanwhile, personal losses related to poor health can be quantified by the earlier appearance of disease, resulting in a decreased quality of life [6,7] and more days of life lost to poor health [8]. Possibly the most cost-effective measure an individual can employ to maintain or improve their health is exercise, as a lack of exercise has been found to be the primary cause of most chronic diseases [9,10,11]. Indeed, the importance of exercise for health is so well known that the motto Exercise is Medicine^®^ has become a global health initiative adopted by organizations such as the American College of Sports Medicine (ACSM) and Exercise and Sports Science Australia (ESSA).

Given the importance of exercise for maintaining or improving health, activity recommendations exist for the general public in countries around the world. Specifically, organizations such as the World Health Organization (WHO), Centers for Disease Control and Prevention (CDC), National Health Service (NHS), and Department of Health and Aged Care (DHAC) recommend adults 18/19–64 years of age perform at least 120 min of moderate-intensity aerobic activity [12,13,14,15] or at least 60–75 min of vigorous-intensity aerobic activity per week [12,13,14,15]. Furthermore, each of these organizations recommend adults 18/19–64 years of age perform muscle-strengthening activities for all major muscle groups at least 2 days per week [12,13,14,15].

Possibly due to the low proportion of the general public who meet the minimum activity recommendations of organizations such as the WHO, CDC, NHS, and DHAC, governments have funded alternative activity interventions in an attempt to increase the activity of the general public. For example, the Australian Government via Queensland Health has provided AUD 4.3 million dollars in funding [16] to grow the popularity of the 10,000 Steps Program [17], despite the 10,000 Steps Program deviating from the activity recommendations of established organizations. The Australian government is also funding the My health for life campaign, which prominently displays the phrase, “no hard training or diets”, in capital letters and bold font on their homepage [18]. Collectively, governments appear to understand the importance of increasing activity levels in the general public to improve health but send inconsistent activity messages to the general public, which may hinder activity participation and adherence.

As a result, we examined the public-facing documentation of the WHO, which aims to improve public health worldwide, the CDC which aims to improve the health of Americans, the NHS, which aims to improve the health of individuals in the United Kingdom, the DHAC, which aims to improve the health of Australians, and the 10,000 Steps Program, which may be the most popular activity recommendation worldwide. We examined the following: (1) the terminology used to describe activity; (2) the recommendations regarding activity intensity; (3) if a distinction was made between physical activity and exercise; (4) if strength activities were recommended; and (5) the weekly time required to complete the minimum activity recommendations for moderate-intensity aerobic activity. We found the activity messages presented to the general public lack clarity and, in some instances, provide conflicting recommendations regarding the activity required to maintain or improve health. More care should be taken to provide a consistent, clear, and direct message to the general public regarding the type and intensity of activity required to maintain or improve health, as making activity recommendations consistent and easier to understand may help increase activity levels.

## 2. Activity Recommendations

Table 1 displays the similarities and differences in activity recommendations provided to the general public. The first discrepancy between the activity recommendations occurs in the terminology used to describe activity. The semantics of the various word choices used to describe the recommended activity to be performed by the general public may seem trivial but it adds unnecessary confusion to public health messages. The WHO uses aerobic activity [12,19] and exercise [12], which causes confusion as exercise can be anaerobic. The CDC uses aerobic activity [13]. The NHS uses activity and exercise [14], suggesting that activity can be aerobic or anaerobic. The DHAC [15] uses physical activity, which can be aerobic or anaerobic. The 10,000 Steps Program recommends taking 10,000 steps per day [17]. The ambiguity in activity recommendations, which are all implied to be aerobic activity, make it difficult for experts in the field to understand what is expected regarding activity recommendations, yet the general public is supposed to have the ability to interpret and apply these recommendations.

There is more consistency regarding the differentiation between moderate and vigorous intensity aerobic activity, as the WHO [12], CDC [13], NHS [14], and DHAC [15] each differentiate between moderate and vigorous intensity activity while the 10,000 Steps Program does not [17]. However, no intervention makes the distinction between physical activity and exercise. Physical activity is defined as any bodily movement produced by skeletal muscle that results in energy expenditure and is characteristically performed in the most efficient manner possible with little regard to physical fitness, as the activity is often performed with energy conversation as a goal [20]. For example, most people will select the easiest walking path, which is evidenced by the widespread use of elevators and escalators. Furthermore, when carrying a child, the emphasis is not on increasing muscle strength but on completing the task as effortlessly as possible. Alternatively, exercise is a subcategory of physical activity and is defined as planned, structured, repetitive, and purposive in an attempt to maintain or improve one or more component of fitness [20]. For example, an individual schedules time to jog three days per week to improve their aerobic capacity. Despite the distinction between physical activity and exercise, none of the government-supported activity recommendations examined make the distinction between physical activity and exercise; yet, we wonder why exercise participation in the general public is low despite the known beneficial effects of exercise on health [19,21,22].

The WHO [12], CDC [13], NHS [14], and DHAC [15] each recommend a strength component in their recommendations, while the 10,000 Steps Program does not [17]. Given the importance of strength training for bone health [23], extending the duration of life [24,25], extending the amount of time individuals can perform activities of daily living [26], and mitigating the effects of sarcopenia [27,28], it is not ideal for the 10,000 Steps Program to ignore the strength component of activity recommendations. However, researchers have previously noted the strength component of government-supported activity recommendations already tends to be overlooked [24,25,29], evidenced by the amount of activities counted as strength activities that are unlikely to maintain or improve the musculoskeletal system of the general public (e.g., tai chi, gardening, household tasks that involve lifting, lifting and carrying children).

The weekly time required to complete the minimum activity recommendations of moderate-intensity aerobic activity is consistent between the WHO [12], CDC [13], NHS [14], and DHAC [15], at 2.5 h per week. The 10,000 Steps Program does not provide a minimum time requirement for moderate-intensity activity [17], but it is possible to quantify the amount of weekly time required to walk 10,000 steps per day. We could not directly find the stride length used by the 10,000 Steps Program to determine the expected distance covered in 10,000 steps, but we utilized the 10,000 Steps Program list of walking pathways in Rockhampton [30] to determine that the 10,000 Steps Program uses an average stride length of 0.80 m. Consequently, we infer the 10,000 Steps Program assumes individuals who take 10,000 steps per day should walk 8 km per day, calculated as 10,000 steps · 0.80 m per step = 8000 m or 8 km. Once the distance of taking 10,000 steps was established, we quantified the expected time to complete 10,000 steps per day using the average walking speeds obtained from a meta-analysis in which the authors quantified the walking speed of individuals across the lifespan [31]. The minimum weekly time required to complete 10,000 steps per day is notably higher (range: 10.85 h per week for 40–49 year olds to 11.62 h per week for 60–69 year olds) than the minimum 2.5 h per week time requirement for weekly moderate intensity activity, as suggested by the WHO [12], CDC [13], NHS [14], and DHAC [15] (Table 2).

An argument can be made in which the difference in time needed to reach the minimum weekly time requirement of moderate-intensity activity between the 10,000 Steps Program and the WHO, CDC, NHS, and DHAC would be less dramatic if one were to find the average steps a person accumulates per day (i.e., discretionary steps) and only count the time required to take the additional steps required to reach 10,000 steps per day. In that regard, a meta-analysis, conducted by Bohannon, consisting of 6199 participants across 42 studies reported an average daily step count of 9448 (95% CI = 8899–9996) [31]. Bohannon also reported the results by age category, in which the daily step count results were reported in individuals <65 years of age and individuals ≥65 years of age. Individuals <65 years of age were reported to accumulate an average daily step count of 9797 (95% CI = 9216–10,337), representing 4899 participants. It was not until Bohannon examined the daily step count of individuals ≥65 years of age, representing 1202 participants, that the daily step count meaningfully deviated from 10,000 steps, with the author reporting a daily step count of 6565 (95% CI = 4897–8233) [31]. Moreover, Tudor-Locke et al. [36] utilized the 2005–2006 National Health and Nutrition Examination Survey (NHANES), in which the daily step count was assessed using accelerometer-determined steps per day (ActiGraph AM-7164) in 3744 participants ≥20 years of age and participants were reported to accumulate 9676 ± 107 steps per day. As a result, it can be argued that a noteworthy number of individuals <65 years of age are meeting the 10,000 steps recommendation without effort; this implies the 10,000 steps recommendation for the attainment of moderate intensity activity may only be practical for those ≥65 years of age. Nevertheless, Table 3 presents a theoretical example of the intentional walking time required to take 10,000 steps, given a discretionary step count of 1000 to 9000 steps per day in 1000 step increments. Individuals taking 7000 discretionary steps per day or less exceed the minimum time recommendation of weekly moderate-intensity activity (2.5 h per week) suggested by the WHO [12], CDC [13], NHS [14], and DHAC [15] when walking 10,000 steps per day.

## 3. Questions Arising from the Activity Messages Being Sent to the General Public

Upon analyzing the activity messages governments are sending to the general public, several key questions emerged. The first being, given the growing prevalence of poor health in the general public, do the recommendations of the WHO, CDC, NHS, or DHAC provide enough of a physical stimulus to maintain or improve health in the general public? In the same vein, given the notoriety of the 10,000 Steps Program [16], is completing 10,000 steps per day enough of a physical stimulus to maintain or improve health in the general public?

A potential concern regarding the efficacy of the activity recommendations of the WHO, CDC, NHS, and DHAC is the apparent fallacy that a vigorous walking pace is required to meet the threshold of moderate-intensity activity (3.0 to <6.0 METs) [37]. The results of the Bohannon and Andrews [33] meta-analysis suggest moderate intensity activity is achieved with a standard (i.e., mean) walking pace by adults. In Table 2, we show a MET level >3.0 is achieved by 20–29 year olds, 40–49 year olds, and 60–69 year olds walking at standard speeds for individuals in their age cohort [33]. These findings suggest vigorous intensity walking is not required to meet the MET requirement of moderate intensity activity.

This ultimately leads to the following question: is walking, which has been demonstrated to occur in the most energy efficient manner possible [38,39], the ideal way to maintain or improve health in the general public? This question is pertinent, as health in the general public continues to degrade and it is well established that more challenging activities yield greater health benefits [21,22]. For example, Booth et al. [40] made a compelling argument regarding the importance of an individual’s lifetime peak bone mineral density, VO_2_ max, and skeletal muscle power in delaying physical frailty (i.e., bone mineral density and skeletal muscle strength) and a lack of vigor (i.e., VO_2_ max), thus increasing the likelihood of dying at an older age. In this regard, peak bone mineral density, peak skeletal muscle power, and peak VO_2_ max cannot be optimized by walking. Moreover, it is impractical for most healthy individuals to improve their aerobic capacity by walking, and the individuals most likely to increase their aerobic capacity through walking would be those who find walking to be challenging, such as diseased populations, populations with extremely low levels of fitness, and older adults (≥65 years); it would not be the general public to which health promotion messages such as those being discussed are targeted.

Our theory regarding walking not being physically demanding enough to maintain or improve health in the general public has support in research, as a systematic review on the effects of low-intensity activity on the risk factors of cardiovascular disease [41] reported no improvements in body mass, waist circumference, body mass index (BMI), glucose, insulin, HbA1c, total cholesterol, high-density lipoprotein (HDL) cholesterol, low-density lipoprotein (LDL) cholesterol, VO_2_ max, resting heart rate, C-reactive protein (CRP), interleukin-6, or tumor necrosis factor alpha (TNF-alpha) in inactive populations, populations with a medical condition or healthy populations. The only improvements reported were for systolic blood pressure in inactive populations (three of five studies reported an improvement) and populations with a medical condition (two of three studies reported an improvement), and for diastolic blood pressure in populations with a medical condition (two of three studies reported an improvement). Even a systematic review and meta-analysis that found walking to be a safe and effective intervention to improve the health of the general public [42] actually produced results that were in agreement with the review of Batacan et al. [41]. Specifically, in a systematic review and meta-analysis conducted by Hanson and Jones [42], the mean age of the participants was 58 years. Moreover, studies included in the analysis utilized participants with a range of health conditions, including arthritis, dementia, cognitive impairment, Parkinson’s disease, mental health issues, diabetes, fibromyalgia, overweight, and obesity. Viewed comprehensively, the results from the Hanson and Jones systematic review and meta-analysis [42] are in agreement with the systematic review and meta-analysis of Batacan et al. [41], which reported that the positive health effects of walking or other forms of low-intensity activity are most likely to occur in older, inactive, or diseased populations.

## 4. Theoretical Path Forward

The importance of encouraging the general public to partake in some form of activity in an attempt to maintain or improve health is unquestioned. However, there are inconsistencies in the government-supported messages designed to encourage the general public to perform activities that will maintain or improve their health. Future research must be conducted with the aim of helping government-supported organizations develop a consistent, clear, and direct message regarding the dissemination of public health activity recommendations. In this regard, we provide several recommendations that help guide future research and potentially guide future practice.

First, public health activity messages should be consistent, clear, and direct. It is not logical for an American to have activity recommendations that differ from an individual residing in the United Kingdom, or an Australian. In order to provide a consistent activity recommendation, consensus must be reached regarding the minimal amount of activity, considering the type (i.e., mode), time, and intensity, that a sedentary individual must perform to meaningfully improve their health on a defined health outcome such as all-cause mortality.

Second, the current activity recommendations examined in this review are for 18/19–64 year olds (WHO [12], CDC [13], NHS [14], DHAC [15]), or throughout life (10,000 Steps Program [17]). The physiological capabilities vary greatly between an 18/19 year old and a 64 year old. As a result, the activity levels required to maintain or improve health in each of these age groups should differ. For example, a meta-analysis [43] comprising 15 studies and 47,471 adult participants reported individuals <60 years of age need to accumulate 8000–10,000 steps per day to optimally reduce their risk of mortality (i.e., gain no additional benefit from accumulating more steps), while individuals ≥60 years of age only need to accumulate 6000–8000 steps per day to optimally reduce their risk of mortality. Future research must be conducted to identify more appropriate age groups for adult activity recommendations, to identify the barriers and enablers that influence different age groups regarding activity participation, and identify how different age groups interpret public health activity recommendations.

Third, consistent terminology needs to be used. Among the activity recommendations examined in this review, activity; implied to be aerobic activity, is referred to as activity (NHS [14]), physical activity (DHAC [15]), aerobic activity (WHO [19], CDC [13]), and exercise (WHO [12], NHS [14]). Inconsistent terminology (i.e., the use of two different terms) is also used to describe aerobic activity by the same organization (the WHO uses aerobic activity and exercise [12]; the NHS uses activity and exercise [14]), or taking 10,000 steps per day (10,000 Steps Program [17]). Ambiguity concerning the nomenclature used to prescribe aerobic activity needs to be resolved. Future research should determine the word most likely to promote adherence to aerobic activity recommendations and that word should be used across all recommendations.

Fourth, the definitions of moderate and vigorous intensity activity need to be simplified and presented in a consistent, clear, and direct manner that can be easily applied by the general public. Current recommendations define moderate intensity activity as 3.0 to less than 6.0 METs (CDC [34]), being able to talk but not sing during the activity (NHS [14]), or do not explicitly provide a definition (WHO [12] and DHAC [15]) in documentation designed for the general public (i.e., the table of their activity recommendations). Current recommendations define vigorous intensity activity as greater than 6.0 METs (CDC [34]), not being able to say more than a few words without taking a breath (NHS [14]), or do not explicitly provide a definition (WHO [12] and DHAC [15]) in documentation designed for the general public (i.e., the table of their activity recommendations). Of the current recommendations, the NHS is likely taking the best approach, as the recommendations regarding moderate and vigorous intensity activity can be easily applied by the general public. Future research should determine if the method employed by the NHS is the best approach to defining moderate and vigorous intensity activity, or if activity intensities should be prescribed by providing a target heart rate range, given the popularity of smart watches.

Fifth, the general public should be better informed about the importance of resistance exercise. Resistance exercise must be a component of activity recommendations for the general public, enabling them to receive the maximal benefit from activity. Resistance exercise can improve aerobic capacity [44] as well as the components of health that cannot be improved with aerobic activity, such full-body improvements in bone mineral density [45]. Research has also found better health benefits in individuals partaking in aerobic and resistance activity rather than aerobic activity alone [46,47]. Moreover, an increasing body of evidence emphasizes the importance of resistance activity over aerobic activity for older adults (>60 years) as the requirements for maintaining activities of daily living (e.g., grocery shopping, ability to stand from lying or sitting) are more dependent on muscle strength and balance than aerobic capacity [48].

Current recommendations from the WHO [12], CDC [34], NHS [14], and DHAC [15] present strength activities as an afterthought and either do not provide suggestions for strength activities (WHO [12]), provide suggestions that are unlikely to improve strength in the general population (CDC [34], NHS [14], DHAC [15]) or completely ignore the strength component (10,000 Steps Program [17]). Future recommendations should make the resistance component of activity recommendations more prominent to accurately reflect the importance of resistance activity on health. Moreover, activities presented as satisfying the strength component should only be suggested if the activities will maintain or improve strength in the age cohorts for which they are being recommended (e.g., Tai Chi is unlikely to improve the strength or bone mineral density of a college-aged adult). It would also be advisable to present strength activities as body weight exercises that can be performed by individuals at different levels of strength. For example, push-ups can be presented as modified push-ups, incline push-ups, push-ups, and decline push-ups to suit individuals with different levels of upper-body strength. Suggesting body weight strength activities would enable the general public to satisfy the strength component of activity recommendations at no economic cost and without having to leave their home.

Table 4 provides a theoretical example of how activity recommendations may be improved in the future. The table is a starting point, and future research will help to create better tables and/or infographics that can be presented to the general public. Activity recommendations aim to help increase the activity levels of the general public. Sending a clear and direct message to the general public will help the general public understand the message, but just knowing the message will not necessarily result in increased adherence. Indeed, we previously found that individuals who were aware of healthy eating practices chose to consume an unhealthy diet [49]. Research has also suggested that the most effective way of influencing behavior is modifying the environment [50]. Therefore, if governments want individuals to walk more, they should install sidewalks. If governments want to encourage individuals to cycle more, they should install bike lanes. If governments truly want to encourage the general public to be more active, improving the consistency and clarity of activity recommendations is only the first step. Governments must also create environments that promote activity.

## 5. Conclusions

The government-supported activity recommendations that are in place to improve the health of the general public are sending mixed messages regarding the type and amount of activity required to maintain or improve health. The positive effects of activity on health are unquestioned, yet a large proportion of the general public are not meeting government-supported activity recommendations. Part of the issue may stem from the general public not being aware of or being confused by the conflicting government-supported activity recommendations. For example, a qualitative study conducted on 15 older adults (minimum age requirement ≥65 yr; mean age: 70.3 ± 3.3 yr) in the United Kingdom found that none of the participants were aware of the current strength training guidelines and reported walking as one of the most common activities they perform to build strength [29]. Currently, government-supported activity recommendations are difficult for experts in the field to understand, so it is unlikely their messages are being understood by their target audience, the general public.

During our review of the literature we noted several aspects of government-supported activity messages that should be improved. (1) Government-supported public health activity messages should be clear, consistent, and direct. (2) We understand government-supported activity recommendations are meant to be broad, but there are vast differences between younger (18/19 year) and older (64 year) individuals regarding the physiological stimulus required to maintain or improve health, their preferred activities, and barriers and enablers regarding activity participation. As a result, it makes little sense to provide the same activity recommendations for individuals of these ages. (3) Consistent terminology should be utilized within and across recommendations. For example, when describing aerobic activity, government-supported activity recommendations have utilized the terms activity, aerobic activity, physical activity, exercise, and taking 10,000 steps per day. The use of varied terminology is not sending a specific, clear, or direct message to the general public about the activity they are being recommended to perform. (4) Definitions for moderate and vigorous intensity activity need to be simplified and presented in a manner that can be easily applied by the general public. For example, METs are currently the most commonly reported measure used to determine moderate or vigorous intensity activity. METs are not applicable to the general public. (5) The general public need to be better informed about the importance of resistance activity. (6) Regarding resistance activity, evidence-based activities that have been demonstrated to improve the musculoskeletal system should be recommended in government-supported activity messages, as some of the activities currently recommended are not able to improve the musculoskeletal system of the general public. Future research must be conducted to determine how to best overcome the issues noted in our review, as the implementation of a consistent, clear, and direct message to the general public using evidence-based practice may empower individuals to become more active, thereby improving their health and reducing the global economic burden of physical inactivity.

## Figures and Tables

**Table 1:** Similarities and differences in activity recommendations provided to the general population.

Intervention	Terminology to Describe “Aerobic” Activity	Differentiation Between Moderate and Vigorous Intensity “Aerobic” Activity	Distinction Between Physical Activity and Exercise	Strength Component	Activities Satisfying the Strength Component
WHO	Aerobic activity [12,19], and exercise [12]	Yes	No	Yes	Not readily specified [19]
CDC	Aerobic activity [13]	Yes	No	Yes	Lifting weights, resistance bands, body weight exercises (e.g., push-ups), heavy gardening, some forms of yoga [13]
NHS	Activity and exercise [14]	Yes	No	Yes	Carrying heavy shopping bags, yoga, Pilates, tai chi, lifting weights, body weight exercise, heavy gardening, wheeling a wheelchair, lifting and carrying children [14]
DHAC	Physical activity [15]	Yes	No	Yes	Push-ups, pull-ups, squats or lunges, lifting weights, household tasks that involve lifting, carrying, or digging [15]
10,000 Steps Program	Take 10,000 steps per day [17]	No	No	No	Not applicable

WHO = World Health Organization, CDC = Centers for Disease Control and Prevention, NHS = National Health Service, DHAC = Department of Health and Aged Care.

**Table 2:** Time and energy required to meet the weekly minimum recommendation of moderate- or vigorous-intensity activity in the public health activity recommendations examined in this review.

Aerobic Activity Recommendation	Time to Achieve Minimum Goal Per Week	Mean Speed (km/h)	Relative VO_2_ (mL/kg/min)	METs	Weekly Caloric Expenditure (Kcal)
*20–29 Years Old, 80 kg Individual, Walking or Jogging on Level Ground (0% Grade)*					
**10,000 Steps Program** **(Walk 8 km)**	11.50 h	4.89	18.99	5.40	5240
**WHO, CDC, NHS, DHAC (Walk)**	2.50 h	4.89	18.99	5.40	1139
**CDC (Vigorous Walk/Jog)**	1.00 h	8.05	28.99	8.28	696
**WHO, NHS, DHAC (Vigorous Walk/Jog)**	1.25 h	8.05	24.25	8.28	870
*40–49 Years Old, 80 kg Individual, Walking or Jogging on Level Ground (0% Grade)*					
**10,000 Steps Program** **(Walk 8 km)**	10.85 h	5.16	19.84	5.67	5166
**WHO, CDC, NHS, DHAC (Walk)**	2.50 h	5.16	19.84	5.67	1190
**CDC (Vigorous Walk/Jog)**	1.00 h	8.05	28.99	8.28	696
**WHO, NHS, DHAC (Vigorous Walk/Jog)**	1.25 h	8.05	28.99	8.28	870
*60–69 Years Old, 80 kg Individual, Walking or Jogging on Level Ground (0% Grade)*					
**10,000 Steps Program** **(Walk 8 km)**	11.62 h	4.82	18.76	5.36	5233
**WHO, CDC, NHS, DHAC (Walk)**	2.50 h	4.82	18.76	5.36	1126
**CDC (Vigorous Walk/Jog)**	1.00 h	8.05	28.99	8.28	696
**WHO, NHS, DHAC (Vigorous Walk/Jog)**	1.25 h	8.05	28.99	8.28	870

The time needed to walk 10,000 steps was quantified by converting 10,000 steps into distance in km. The 10,000 Steps Program appears to assume a mean stride length of 0.8 m [32], yielding 8 km per 10,000 steps. The man walk speed was determined for each age groups, as follows: 20–29 yr = 4.89 km/h, 40–49 yr = 5.16 km/h, and 60–69 yr = 4.82 km/h [33]. Vigorous walk/jog speed was determined using the vigorous activity speed recommendation of the CDC, in accordance with the U.S. Department of Health and Human Services [34]. The relative VO_2_, METs, and kcals were calculated using the American College of Sports Medicine formula [35]. The energy expenditure was estimated using the American College of Sports Medicine formula [35]. For all calculations, an 80 kg male, moving on a 0 grade ground, was utilized. WHO = World Health Organization, CDC = Centers for Disease Control and Prevention, NHS = National Health Service, DHAC = Department of Health and Aged Care, VO_2_ = volume of oxygen, METs = metabolic equivalents.

**Table 3:** The time needed for a 20–29 year old to walk the steps required to reach 10,000 steps per day, assuming a discretionary step count of 1000 to 9000 steps per day in 1000 step increments.

Discretionary Steps	Additional Steps Required to Reach 10,000	Stride Length	Distance Walked in Additional Steps	Walk Speed	Additional Time Per Day to Reach 10,000 Steps	Additional Time Per Week to Reach 10,000 Steps Per Day
1000	9000	0.8 m	7.2 km	4.89 km/h	1.47 h	10.29 h ^†^
2000	8000	0.8 m	6.4 km	4.89 km/h	1.31 h	9.17 h ^†^
3000	7000	0.8 m	5.6 km	4.89 km/h	1.15 h	8.05 h ^†^
4000	6000	0.8 m	4.8 km	4.89 km/h	0.98 h	6.86 h ^†^
5000	5000	0.8 m	4.0 km	4.89 km/h	0.82 h	5.74 h ^†^
6000	4000	0.8 m	3.2 km	4.89 km/h	0.65 h	4.55 h ^†^
7000	3000	0.8 m	2.4 km	4.89 km/h	0.49 h	3.43 h ^†^
8000	2000	0.8 m	1.6 km	4.89 km/h	0.33 h	2.31 h
9000	1000	0.8 m	1.25 km	4.89 km/h	0.26 h	1.82 h

The time needed to walk 10,000 steps was quantified by converting 10,000 steps into distance in km. The 10,000 Steps Program appears to assume a mean stride length of 0.8 m [32], yielding 8 km per 10,000 steps. The mean walk speed of 4.89 km/h for a 20–29 yr old was determined from a meta-analysis [33]. ^†^ Represents walk times that exceed the minimum weekly threshold of 2.5 h, as recommended by the World Health Organization, Centre for Disease Control and Prevention, National Health Service, and Department of Health and Aged Care.

**Table 4:** Theoretical table of a clear and direct message that should be easily understandable across cultures and varied educational backgrounds.

Age	Level 1: Sedentary	Level 2: Minimally Physically Active	Level 3: Physically Active	Level 4: Physically Healthy
**18 to less than 45 years**	Walk or perform aerobic activities that are less intense than walking and equate to <TBD minutes per week	Walk and engage in additional aerobic activities more intense than walking (e.g., jogging, cycling) for ≥TBD minutes per week	Walk ≥TBD minutes per week AND perform aerobic activities in which you will not be able to say more than a few words without taking a breath for ≥TBD minutes per week AND resistance training: TBD sets for each major muscle group 2 days per week	Walk ≥TBD minutes per week AND perform aerobic activities in which you will not be able to say more than a few words without taking a breath for ≥TBD minutes per week AND resistance training: TBD sets for each major muscle group 2 days per week
**45 to less than 65 years**	Walk or perform aerobic activities that are less intense than walking and equate to <TBD minutes per week	Walk and engage in additional aerobic activities that are more intense than walking (e.g., jogging, cycling) for ≥TBD minutes per week	Walk ≥TBD minutes per week AND perform aerobic activities in which you will not be able to say more than a few words without taking a breath for ≥TBD minutes per week AND resistance training: TBD sets for each major muscle group 2 days per week	Walk ≥TBD minutes per week AND perform aerobic activities in which you will not be able to say more than a few words without taking a breath for ≥TBD minutes per week AND Resistance training: TBD sets for each major muscle group 2 days per week
**65 years or older**	Walk or perform aerobic activities that are less intense than walking and equate to <TBD minutes per week	Walk and engage in additional aerobic activities that are more intense than walking (e.g., jogging, cycling) for ≥TBD minutes per week	Walk ≥TBD minutes per week AND perform aerobic activities in which you will not be able to say more than a few words without taking a breath for ≥TBD minutes per week AND resistance training: TBD sets for each major muscle group 2 days per week	Walk ≥TBD minutes per week AND perform aerobic activities in which you will not be able to say more than a few words without taking a breath for ≥TBD minutes per week AND resistance training: TBD sets for each major muscle group 2 days per week
*Expected Health Outcomes*				
**Health Indicator**	** 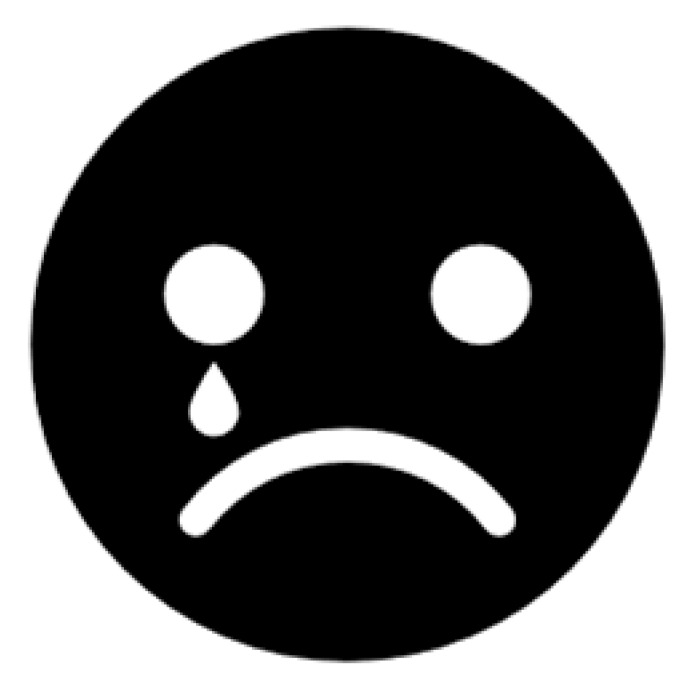 **	** 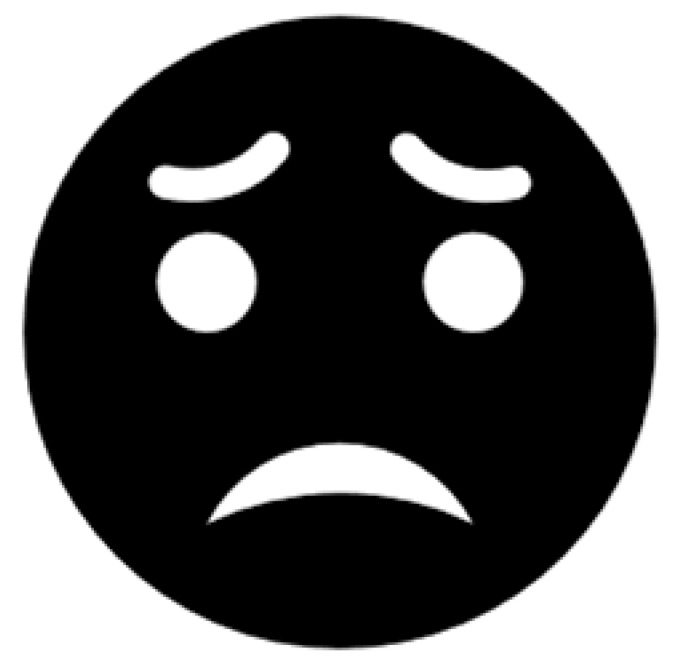 **	** 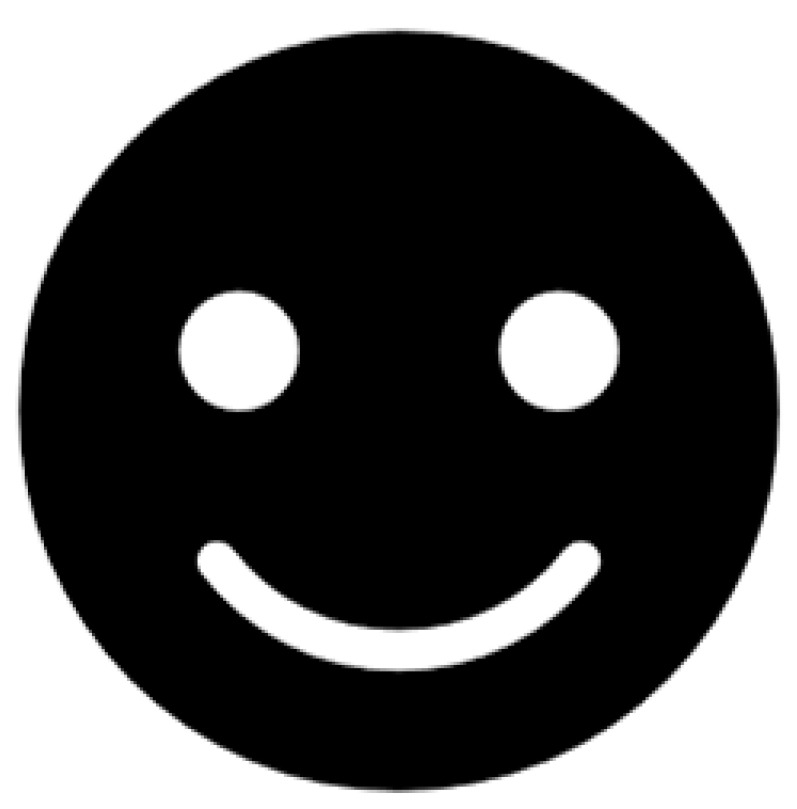 **	** 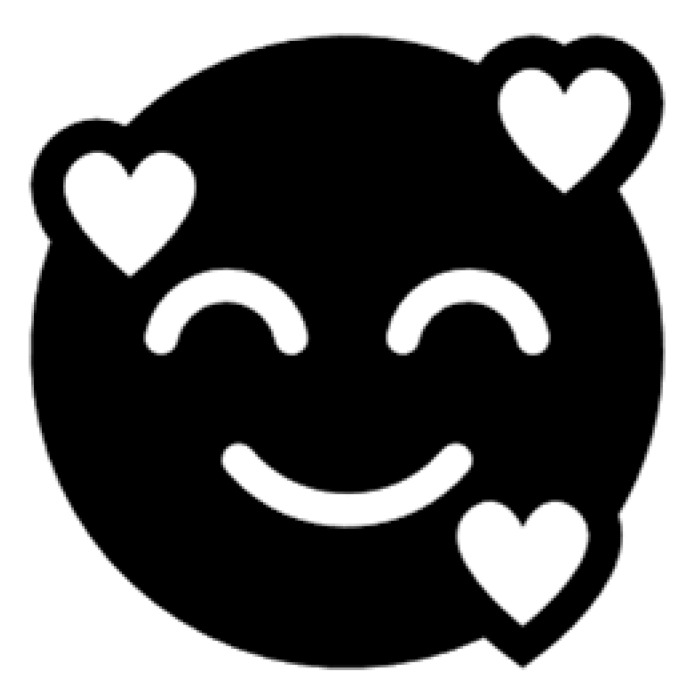 **
**Expected Benefits**	None, you are doing more harm than good	Live longer	Live longer, maintain independence longer, maintain a higher quality of life, improve balance, strength, flexibility, and coordination	Live longer, maintain independence longer, maintain a higher quality of life, improve balance, strength, flexibility, and coordination
**Effect of Your Activity Level on Your Health**	Your lack of activity is killing you	Great start, your activity level is starting to improve your health	Good job, your activity level is greatly improving your health	Great job, you are maximizing the health benefits of activity

Table assumptions: (1) Future research finds these age groupings to be appropriate to optimize activity recommendations to the general public. (2) No intensity description regarding breathing was provided for moderate-intensity aerobic activity, as research suggests that the average walking speeds equate to moderate-intensity activity [33], as defined by a MET level of 3.0 to less than 6.0 METs [34]. (3) Vigorous-intensity aerobic activity was described using a description of breathing utilized by the National Health Service, assuming future research finds this description better than using a target heart rate range. (4) Activity recommendations for each age group are theoretical. Future research must clearly define the activity recommendations for each age group and activity level. (5) The resistance training component can be accomplished with body weight exercises. TBD = To be determined by future research.

## Data Availability

No new data were created or analyzed in this study. Data sharing is not applicable to this article.

## References

[1] Australian Institute of Health and Welfare (2022). How Do We Measure the Cost of Disease?. https://www.aihw.gov.au/getmedia/1c2bf0de-c981-4f04-971d-47dfc8867ff0/bod_expenditure_factsheet.pdf.aspx#:~:text=The%20human%20cost%20of%20particular,years%20of%20healthy%20life%20lost'.

[2] Callander E.J., Fox H., Lindsay D. (2019). Out-of-pocket healthcare expenditure in Australia: Trends, inequalities and the impact on household living standards in a high-income country with a universal health care system. Health Econ. Rev..

[3] Lee D.W., Lee J., Kim H.R., Kang M.Y. (2021). Health-Related Productivity Loss According to Health Conditions among Workers in South Korea. Int. J. Environ. Res. Public Health.

[4] Zhang W., Bansback N., Anis A.H. (2011). Measuring and valuing productivity loss due to poor health: A critical review. Soc. Sci. Med..

[5] National Health Service (2024). NHS Productivity. Public Board Paper (BM/24/19(Pu)..

[6] Australian Institute of Health and Welfare (2023). Australian Burden of Disease Study 2023, Summary—Australian Institute of Health and Welfare. https://www.aihw.gov.au/reports/burden-of-disease/australian-burden-of-disease-study-2023/contents/summary.

[7] World Health Organization (2024). Obesity and Overweight. https://www.who.int/news-room/fact-sheets/detail/obesity-and-overweight.

[8] Institution of Health Metrics Evaluation (IHME) (2018). Findings from a Global Burden Disease Study 2017.

[9] Booth F.W., Roberts C.K., Laye M.J. (2012). Lack of exercise is a major cause of chronic diseases. Compr. Physiol..

[10] Ruegsegger G.N., Booth F.W. (2018). Health Benefits of Exercise. Cold Spring Harb. Perspect Med..

[11] Booth F.W., Gordon S.E., Carlson C.J., Hamilton M.T. (2000). Waging war on modern chronic diseases: Primary prevention through exercise biology. J. Appl. Physiol..

[12] World Health Organisation (2024). Physical Activity. https://www.who.int/news-room/fact-sheets/detail/physical-activity.

[13] Centers for Disease Control and Prevention (2023). Physical Activity for Adults: An Overview. https://www.cdc.gov/physical-activity-basics/guidelines/adults.html#:~:text=Physical%20activity%20is%20one%20of,muscle%2Dstrengthening%20activity%20each%20week.

[14] National Health Service (2021). Physical Activity Guidelines for Adults Aged 19 to 64. https://www.health.gov.au/topics/physical-activity-and-exercise/physical-activity-and-exercise-guidelines-for-all-australians/for-adults-18-to-64-years#:~:text=Recommendations,mowing%20the%20lawn%20or%20swimming.

[15] Australian Government Department of Health and Aged Care (2021). Physical Activity and Exercise Guidelines for All Australian: For Adults (18 to 64 Years). https://www.health.gov.au/topics/physical-activity-and-exercise/physical-activity-and-exercise-guidelines-for-all-australians/for-adults-18-to-64-years.

[16] Vandelanotte C., Van Itallie A., Brown W., Mummery W.K., Duncan M.J. (2020). Every step counts: Understanding the success of implementing the 10,000 Steps Project. Stud Health Technol. Inform..

[17] 10,000 Steps—About Us. https://www.10000steps.org.au/articles/10000-steps-program/about-us/.

[18] My Health for Life. https://www.myhealthforlife.com.au.

[19] Bull F.C., Al-Ansari S.S., Biddle S., Borodulin K., Buman M.P., Cardon G., Carty C., Chaput J.-P., Chastin S., Chou R. (2020). World Health Organization 2020 guidelines on physical activity and sedentary behaviour. Br. J. Sports Med..

[20] Caspersen C.J., Powell K.E., Christenson G.M. (1985). Physical activity, exercise, and physical fitness: Definitions and distinctions for health-related research. Public Health Rep..

[21] Atakan M.M., Li Y., Kosar S.N., Turnagol H.H., Yan X. (2021). Evidence based effects of high-intensity interval training on exercise capacity and health: A review with historical perspective. Int. J. Environ. Res. Public Health.

[22] Gebel K., Ding D., Chey T., Stamatakis E., Brown W.J., Bauman A.E. (2015). Effect of moderate to vigorous physical activity on all-cause mortality in middle-aged and older Australians. JAMA Intern. Med..

[23] Hong A.R., Kim S.W. (2018). Effects of resistance exercise on bone health. Endocrinol. Metab..

[24] Dalbo V.J., Czerepusko J.B., Tucker P.S., Kingsley M.I., Moon J.R., Young K., Scanlan A.T. (2015). Not sending the message: A low prevalence of strength-based exercise participation in rural and regional Central Queensland. Aust. J. Rural Health.

[25] Steele J., Fisher J., Skivington M., Dunn C., Arnold J., Tew G., Batterham A.M., Nunan D., O’Driscoll J.M., Mann S. (2017). A higher effort-based paradigm in physical activity and exercise for public health: Making the case for a greater emphasis on resistance training. BMC Public Health..

[26] Mayer F., Scharhag-Rosenberger F., Carlsohn A., Cassel M., Müller S., Scharhag J. (2011). The intensity and effects of strength training in the elderly. Dtsch. Arztebl. Int..

[27] Law T.D., Clark L.A., Clark B.C. (2016). Resistance Exercise to Prevent and Manage Sarcopenia and Dynapenia. Annu. Rev. Gerontol. Geriatr..

[28] Hurst C., Robinson S.M., Witham M.D., Dodds R.M., Granic A., Buckland C., De Biase S., Finnegan S., Rochester L., Skelton D.A. (2022). Resistance exercise as a treatment for sarcopenia: Prescription and delivery. Age Ageing.

[29] Gluchowski A., Bilsborough H., McDermott J., Hawley-Hague H., Todd C. (2022). ‘A Lot of People Just Go for Walks, and Don’t Do Anything Else’: Older Adults in the UK are not aware of the strength component embedded in the Chief Medical Officers’ physical activity guidelines—A qualitative study. Int. J. Environ. Res. Public Health.

[30] National Heart Foundation of Australia Healthy Active by Design Introductory Training Resource. https://www.healthyactivebydesign.com.au/resources/healthy-active-by-design-training-resource.

[31] Bohannon R.W. (2007). Number of pedometer-assessed steps taken per day by adults: A descriptive meta-analysis. Phys. Ther..

[32] Rockhampton Regional Council 10,000 Steps Community Walkways. https://www.10000steps.org.au/office/wagtaildocs/472/RRC_10000_Steps_DL_BROCHURE_FOR_WEBSITE.pdf.

[33] Bohannon R.W., Williams Andrews A. (2011). Normal walking speed: A descriptive meta-analysis. Physiotherapy.

[34] US Department of Health and Human Services (2018). Physical Activity Guidelines for Americans.

[35] Bushman B.A. (2023). Metabolic Calculations Cases. ACSM’S Health Fit J..

[36] Tudor-Locke C., Johnson W.D., Katzmarzyk P.T. (2009). Accelerometer-determined steps per day in US adults. Med. Sci. Sports Exerc..

[37] Centers for Disease Control and Prevention (2017). National Health Interview Survey. https://www.cdc.gov/nchs/nhis/physical_activity/pa_glossary.htm#print.

[38] Selinger J.C., O’Connor S.M., Wong J.D., Donelan J.M. (2015). Humans Can Continuously Optimize Energetic Cost during Walking. Curr. Biol..

[39] Bertram J.E. (2015). Locomotion: Why we walk the way we walk. Curr. Biol..

[40] Booth F.W., Laye M.J., Roberts M.D. (2011). Lifetime sedentary living accelerates some aspects of secondary aging. J. Appl. Physiol..

[41] Batacan R.B., Duncan M.J., Dalbo V.J., Tucker P.S., Fenning A.S. (2015). Effects of light intensity activity on cvd risk factors: A systematic review of intervention studies. Biomed. Res. Int..

[42] Hanson S., Jones A. (2015). Is there evidence that walking groups have health benefits? A systematic review and meta-analysis. Br. J. Sports Med..

[43] Paluch A.E., Bajpai S., Bassett D.R., Carnethon M.R., Ekelund U., Everson K.R., Galuska D.A., Jefferis B.J., Kraus W.E., Lee I.-M. (2022). Daily steps and all-cause mortality: A meta-analysis of 15 international cohorts. Lancet Public Health.

[44] Ozaki H., Loenneke J.P., Thiebaud R.S., Abe T. (2013). Resistance training induced increase in VO_2_max in young and older subjects. Euro. Rev. Aging Phys. Act..

[45] Benedetti M.G., Furlini G., Zati A., Letizia Mauro G. (2018). The Effectiveness of physical exercise on bone density in osteoporotic patients. Biomed. Res. Int..

[46] Atkin S.L., Schroeder E.C., Franke W.D., Sharp R.L., Lee D.-c. (2019). Comparative effectiveness of aerobic, resistance, and combined training on cardiovascular disease risk factors: A randomized controlled trial. PLoS ONE.

[47] An J., Su Z., Meng S. (2024). Effect of aerobic training versus resistance training for improving cardiorespiratory fitness and body composition in middle-aged to older adults: A systematic review and meta-analysis of randomized controlled trials. Arch. Gerontol. Geriatr..

[48] Liu C.J., Latham N.K. (2009). Progressive resistance strength training for improving physical function in older adults. Cochrane Database Syst. Rev..

[49] Dalbo V.J., Hiskens M.I., Teramoto M., Kingsley M.I., Young K.C., Scanlan A.T. (2017). Residents of Central Queensland, Australia are aware of healthy eating practices but consume unhealthy diets. Sports.

[50] Kwasnicka D., Dombrowski S.U., White M., Sniehotta F. (2016). Theoretical explanations for maintenance of behaviour change: A systematic review of behaviour theories. Health Psychol. Rev..

